# Behavioral and Psychological Correlates of Depressive Symptoms in Korean Women: Attenuation of the Perceived Body Size Association After Adjustment

**DOI:** 10.3390/healthcare14172859

**Published:** 2026-09-04

**Authors:** Sujung Lee, Dae-Soon Son, Jae-In Kim, Jungmin Lee

**Affiliations:** 1 School of Nursing, Research Institute of Nursing Science, Hallym University, Chuncheon 24252, Republic of Korea; sujunglee@hallym.ac.kr; 2 Smart-Healthcare Convergence, College of Natural Sciences, Hallym University, Chuncheon 24252, Republic of Korea; biostat@hallym.ac.kr; 3 Institute of New Frontier Research, Division of Big Data and Artificial Intelligence, College of Medicine, Hallym University, Chuncheon 24252, Republic of Korea; 4 Hallym AI-BioHealth R&BD Center, Research Institute of Medical-Bio Convergence, Hallym University, Chuncheon 24252, Republic of Korea; 5 Department of Data Science and Data Science Convergence Research Center, Hallym University, Chuncheon 24252, Republic of Korea; 6 Department of Physiology, Neurology, Hallym University, Chuncheon 24252, Republic of Korea; qzk12344@gmail.com

**Keywords:** perceived body size, depression, women, psychosocial factors, age-specific correlates

## Abstract

**Aim**: This study addressed two research questions. First, which sociodemographic, behavioral, and psychological factors are independently associated with elevated depressive symptoms among Korean women aged 20–59 years? Second, does subjective perception of one’s own body size retain an independent association with depressive symptoms after these factors are taken into account, and does this pattern differ between younger and midlife women? The primary outcome was elevated depressive symptoms, defined as a PHQ-9 score of 5 or above. **Design**: This cross-sectional study used pooled data from the four Korea National Health and Nutrition Examination Survey cycles in which the PHQ-9 was administered (2014, 2016, 2018, and 2020). All estimates incorporate the complex survey design and therefore refer to the national population of Korean women aged 20–59 years. **Methods**: We analysed data from Korean women aged 20–59 years who completed the PHQ-9 (*n* = 7595). Elevated depressive symptoms were defined as a PHQ-9 score of 5 or above, indicating mild or greater symptomatology. Design-based multivariable logistic regression models were constructed in sequential blocks: demographic variables (Model 1); behavioural and psychological factors added (Model 2); and perceived body size, entered as indicator contrasts against average body size, added (Model 3). Analyses were stratified by age group (20–39 and 40–59 years), and age-group interaction terms were fitted in a pooled model to test formally whether associations differed across the female lifespan. **Results**: The weighted prevalence of elevated depressive symptoms (PHQ-9 ≥ 5) was 24.1% (95% CI 22.8–25.3). In bivariate analyses, perceived body size differed significantly between women with and without elevated depressive symptoms (*p* < 0.001). After adjustment for behavioural and psychological factors, neither perceived thinness nor perceived obesity was associated with depressive symptoms in either age group (20–39 years, thin: OR 1.25, 95% CI 0.92–1.69; obese: OR 1.05, 95% CI 0.85–1.30; 40–59 years, thin: OR 1.21, 95% CI 0.84–1.73; obese: OR 0.99, 95% CI 0.80–1.22), and the joint two-degree-of-freedom test was non-significant in both groups. Perceived stress (OR 5.15 and 4.93), poor subjective health (OR 2.11 and 2.55), and lifetime smoking experience (OR 1.75 and 1.79) showed the strongest and most consistent associations in both groups (all *p* < 0.001). Lower household income was associated with depressive symptoms among women aged 40–59 years and heavy episodic drinking among those aged 20–39 years; in formal interaction testing, household income showed only a non-significant trend towards effect modification by age (*p* = 0.060), so the age differences are described rather than established. **Conclusions**: The crude association between perceived body size and depressive symptoms did not persist after adjustment for behavioral and psychological factors. Because perceived stress and subjective health may lie on a causal pathway from body size perception to depressive symptoms, this attenuation is consistent with either confounding or an indirect pathway, and the present cross-sectional design cannot distinguish between them. Perceived stress and subjective health showed the strongest associations across age groups, while lower household income remained an independent correlate in midlife. This finding points to a material condition that individual-level screening cannot by itself address.

## 1. Introduction

Depression is a leading contributor to the burden of disease among women, and its determinants in the Republic of Korea remain incompletely understood, particularly with respect to appearance- and weight-related factors [1]. Although research on women’s health has traditionally emphasized physiological risk factors such as body mass index (BMI), diet, and physical activity [2,3], recent attention has shifted toward psychosocial determinants—especially perceived body size [4]. This construct, defined as an individual’s subjective evaluation of her/his body size or shape, plays a vital role in shaping health-related behaviors, emotional well-being, and quality of life [5]. Importantly, this perception may not always align with objective weight status, and such misalignment can lead to inappropriate or harmful health behaviors [6].

Women are particularly vulnerable to distorted body image due to persistent societal pressures surrounding appearance, thinness, and femininity [7]. Numerous studies have shown that women, more than men, are likely to internalize unrealistic body ideals propagated through media and cultural norms that lead to increased body dissatisfaction and engagement in weight control practices regardless of actual BMI [8,9]. Two types of perceptual misalignment are common: underestimation, which may delay necessary weight management efforts, and overestimation, which can result in unnecessary dieting, psychological distress, and risk of eating disorders [10]. These issues are especially concerning given their association with mental health conditions such as depression and anxiety [11]. A conceptual distinction should be drawn at the outset. Body image is a multidimensional construct encompassing affective, cognitive, evaluative, and perceptual components, of which body dissatisfaction and appearance-related distress are the dimensions most often implicated in depression. The present study measures only one narrow component of this construct: an individual’s categorical appraisal of her own body size. This appraisal is conceptually distinct from dissatisfaction with that body, and the two may diverge, since a woman who perceives herself as heavier than average need not be distressed by that perception. We therefore use the term perceived body size when referring to our own measure, and reserve body image and body dissatisfaction for the broader literature reviewed here.

Despite the growing prevalence of body image concerns, existing research has often failed to account for age-related differences among women. Most studies tend to treat adults as a homogenous group, neglecting that psychosocial and physiological contexts influencing perceived body size can vary significantly between younger and middle-aged women [12,13]. Younger women may be more influenced by peer comparison and media exposure [14], whereas middle-aged women may have body image concerns related to aging, menopause, or metabolic health changes [15]. These age-specific dynamics suggest that the factors shaping perceived body size and its health consequences are not uniform across the female lifespan.

A further question concerns why subjective appraisal diverges from measured status at all, and why that divergence is patterned by socioeconomic position. Health literacy—the capacity to access, appraise, and act upon health information—offers one account. Women differing in education and income differ systematically in their exposure to health information, in the interpretive frameworks they bring to bodily and somatic signals, and in the reference groups against which they calibrate what counts as a normal body or as good health. On this reading, both perceived body size and self-rated health are appraisals partly constituted by informational and socioeconomic position rather than transparent readings of an underlying physical state, which would account for the education and income gradients observed in such measures. This framework also carries an implication relevant to interpretation: health literacy may operate on depressive symptoms through direct and indirect pathways that need not run in the same direction, so that a null association at the level of a single appraisal measure does not preclude structured pathways beneath it.

Integrated research that examines how perceived body size interacts with health behaviors, psychological distress, and biological markers among different age groups is lacking. Thus, whether the same variables influence perceived body size in women during different life stages or whether distinct patterns emerge depending on sociodemographic or physiological contexts remains unclear. A nuanced understanding of these differences is essential to inform tailored, age-appropriate public health interventions aimed at promoting healthy perceived body size and preventing obesity-related comorbidities.

This study addressed these gaps using pooled data from the four Korea National Health and Nutrition Examination Survey (KNHANES) cycles in which the PHQ-9 was administered (2014, 2016, 2018, and 2020). The primary outcome throughout was elevated depressive symptoms, operationalized as a PHQ-9 score of 5 or above. Three research questions were specified. First, which sociodemographic, behavioral, and psychological characteristics are independently associated with elevated depressive symptoms among Korean women aged 20–59 years? Second, does perceived body size retain an independent association with depressive symptoms once behavioral and psychological characteristics are taken into account? Third, do the characteristics associated with depressive symptoms differ between younger women aged 20–39 years and midlife women aged 40–59 years? Given prior evidence that appearance-related concerns are more salient in younger adulthood while socioeconomic and relational conditions become more salient in midlife, we anticipated that perceived body size would be more strongly associated with depressive symptoms among younger women, and that income and household composition would be more strongly associated among midlife women. Biochemical markers were examined descriptively only and were not entered into the multivariable models.

## 2. Materials and Methods

### 2.1. Study Design and Participants

This cross-sectional study used data from the KNHANES 2014, 2016, 2018, and 2020 cycles. The KNHANES is a nationally representative, population-based survey conducted annually by the Korea Disease Control and Prevention Agency. It utilizes a stratified, multistage probability sampling method to ensure representativeness of the Korean population. The survey collects comprehensive data on demographics, lifestyle behaviors, mental and psychological health, and biochemical markers through self-reported questionnaires, health examinations, and laboratory tests.

This study focused on women aged 20–59 years, a critical period of adulthood characterized by transitions in health behaviors and metabolic risk factors. Individuals were included in the study if they had complete data on key variables related to demographics, mental health, and biochemical markers.

The PHQ-9 was introduced to the KNHANES in 2014 and has since been administered in selected survey cycles only. The present analyses therefore draw on the four cycles in which the instrument was fielded (2014, 2016, 2018, and 2020). A single analytic sample is used throughout. Of the 7647 women aged 20–59 years who completed the PHQ-9, 7595 had complete information on all study variables and on the survey design identifiers, and these women constitute the analytic sample. Descriptive statistics, bivariate comparisons, and all three regression models were estimated using this same sample, ensuring consistent denominators across analyses and direct comparability of the sequential models.

### 2.2. Study Variables and Measurements

To comprehensively analyze the factors associated with depressive symptoms in women, this study categorized variables into 4 major domains: demographic factors, lifestyle and behavioral factors, mental and psychological health status, and biochemical markers. These categories were selected based on prior evidence indicating their potential impact on depression among women.

Demographic characteristics including age, residence area, educational level, household income, and household generation were examined as fundamental determinants of health disparities.

Household incomes were categorized into quartiles based on the monthly equivalized household income; lowest 25% first, lower-middle 25% second, upper-middle 25% third, and highest 25% fourth. Throughout the regression analyses, the reference category was the highest income quartile for household income, college or higher for educational level, city (dong) for residential area, and multi-person household for household generation. Among the behavioral variables, the reference category was no lifetime smoking for smoking, no for heavy episodic drinking, yes for muscle-strengthening exercise, good for subjective health status, and low or moderate for perceived stress. For perceived body size, average was the reference category.

Residential area was classified as “city” (dong) or “rural” (eup/myeon) according to administrative districts based on the Population and Housing Census during the sample design stage. Educational levels were categorized as: “high school or lower,” graduates of middle or high school; “associate degree,” graduates of 2–3-year colleges; and “college or higher,” graduates of a 4-year college or graduate school program.

Lifestyle and behavioral factors including current smoking, alcohol consumption, muscle-strengthening exercise, subjective health status, subjective body perception, and BMI. Smoking was assessed with the current smoking status item, and respondents who reported smoking at the time of the survey or having smoked previously were classified as having lifetime smoking experience, with lifelong non-smokers as the reference. This variable therefore captures ever having smoked rather than current smoking, and the proportion reported here is correspondingly higher than the national current-smoking rate for women. Heavy episodic drinking was defined as consuming 10 or more alcoholic drinks on a single occasion, classified as “yes”; consuming fewer than 10 drinks on a single occasion was classified as “no.” This threshold is higher than the standard Korean criterion for binge drinking in women (≥5 drinks per occasion); accordingly, this measure captures a more extreme, heavy episodic drinking pattern and likely yields a conservative prevalence estimate. Muscle-strengthening exercise was assessed with the item asking on how many days during the past week the respondent had performed muscle-strengthening activities such as push-ups, sit-ups, weight training, or the use of resistance equipment. Respondents reporting at least one day per week were classified as “yes,” and those reporting no days as “no.” We adopted a threshold of at least one day per week rather than the two-days-per-week criterion used for the national muscle-strengthening exercise indicator, in order to capture any regular engagement in resistance activity among community-dwelling women, among whom participation is low overall. Results under the national two-day criterion are reported as a sensitivity analysis.

Subjective health status was determined from the responses to the question “How do you usually think your health is?” with “very good” and “good” responses categorized as “GOOD,” and “bad” and “very bad,” categorized as “BAD.” Other responses were categorized as “Average.” Subjective body perception was determined from the responses to the question, “How would you describe your current body type?” with responses “Very thin” and “Slightly thin” defined as “Thin,” “Slightly Obese” and “Very Obese” defined as “Obese,” and “Average” defined as “Average.” BMI was categorized as: <18.5 “underweight,” >18.5 and <23 “normal,” and >23 “obesity.” This single-item measure captures a categorical appraisal of body size and does not assess satisfaction with, or distress about, that body. It is therefore not a measure of body image or body dissatisfaction in the sense used in the psychological literature, and results should be interpreted accordingly. Single-item body size appraisal is nonetheless widely used in population surveys, and its association with weight-control behavior and psychological distress has been documented in large national samples, which supports its use as an indicator of perceptual appraisal even though it cannot substitute for a validated multidimensional instrument.

The Patient Health Questionnaire-9 (PHQ-9) scores were categorized using “None” for scores ≤ 4 and “Yes” for scores ≥ 5. A cut-off of ≥5 identifies mild or greater depressive symptoms; this threshold was selected to capture subclinical as well as clinically relevant symptomatology in a general-population sample, and it should be noted that it is more inclusive than the ≥10 cut-off commonly used to screen for probable major depression. The perceived stress rate is the percentage of participants who responded that they felt “very much” or “a lot” stressed in their daily lives. It is calculated by dividing the number of participants who gave this response by the total number of respondents. The perceived stress rate = (number of participants who felt “very” or “a lot” stressed)/(total number of respondents) × 100.

Biochemical markers, comprising systolic and diastolic blood pressure, high-density lipoprotein cholesterol, fasting blood glucose, triglycerides, and glycated hemoglobin, were assessed solely to characterize the cardiometabolic and glycemic profile of the sample. They were specified in advance as descriptive variables and were not entered into any multivariable model, since the research questions concerned sociodemographic, behavioral, and psychological characteristics. Readers should therefore not expect these markers among the multivariable results reported in Section 3.3.

### 2.3. Statistical Analyses

Regression results are reported as odds ratios with 95% confidence intervals alongside the regression coefficients and standard errors, to facilitate interpretation of effect magnitude. All statistical analyses were performed using the R software version 4.4.0 (R Foundation for Statistical Computing, Vienna, Austria).

Descriptive statistics were used to compare demographic, lifestyle, and behavioral factors, mental and psychological health status, and biochemical characteristics. We compared the presence or absence of elevated depressive symptoms based on cutoff value 5 for PHQ-9 scores.

All analyses accounted for the complex sampling design of the KNHANES in accordance with the raw-data analysis guidelines issued by the Korea Disease Control and Prevention Agency. Integrated sampling weights were constructed by dividing the health-interview and examination weight by the number of pooled survey years, following the across-phase pooling rule specified in the KNHANES user guide, and the variance-estimation stratum and the primary sampling unit were specified as the stratification and clustering variables. Because deleting cases from a complex-survey file discards design information and biases standard errors, women aged 20–59 years with a completed PHQ-9 were analysed as a subpopulation within the full design rather than extracted as a separate file. Variances were estimated by Taylor-series linearisation across 106 strata and 747 primary sampling units, giving 641 design degrees of freedom. Continuous variables are summarised as weighted means with standard deviations and categorical variables as weighted percentages with unweighted counts; group differences were tested with design-based Wald F tests.

To analyze the factors associated with elevated depressive symptoms, we built a multiple logistic regression model and set up 3 models by adding variables in sequential steps. Model 1: covariates were fixed with demographic factors (area of residence + education level + income quartile (household) + household generation); Model 2 additionally adjusted for behavioral and psychological factors (lifetime smoking experience + heavy episodic drinking + muscle-strengthening exercise + subjective health status + perceived stress); and Model 3 added perceived body size to Model 2. All *p*-values were calculated using a 2-sided test, with values < 5% considered significant.

Logistic models were used to predict elevated depressive symptoms, and prediction performance was evaluated using weighted accuracy, sensitivity, specificity, and the area under the receiver operating characteristic curve (AUC), with the classification threshold set at the weighted prevalence within each age group. All three models were estimated on the same analytic sample, so the sequential comparison of their performance is not confounded by a change in sample composition. Four sets of sensitivity analyses were pre-specified. First, perceived body size was entered as indicator contrasts and tested jointly on two degrees of freedom. Second, the outcome was redefined using the PHQ-9 cut-off of 10 or above, which corresponds to the national indicator for depressive disorder. Third, the PHQ-9 total score was modelled as a continuous outcome by design-based linear regression, to establish whether dichotomisation discarded information. Fourth, age-group interaction terms were fitted in a pooled model to test formally whether associations differed across the female lifespan, and muscle-strengthening exercise was re-specified using the national two-days-per-week criterion.

### 2.4. Ethical Considerations

The study was conducted using publicly available, de-identified KNHANES data. Ethical approval was obtained from the Institutional Review Board (IRB) of Hallym University (Approval No.: HIRB-2024-058). All research procedures adhered to the ethical guidelines outlined in the Declaration of Helsinki. This study does not contain any individual-level, identifiable data requiring explicit participant consent for publication.

## 3. Results

### 3.1. General Characteristics

A total of 7595 women aged 20–59 years formed the analytic sample (Table 1). All proportions reported hereafter are weighted for the complex survey design and therefore describe Korean women aged 20–59 years. The age distribution was relatively balanced, with the largest proportion in the age group 40–49 years (27.9%), followed by 50–59 years (25.3%), 30–39 years (24.5%), and 20–29 years (22.3%).

The majority of participants (88.1%) resided in urban areas, whereas 11.9% lived in rural areas; 39.6% had completed college or higher, 20.3% held an associate degree, and 40.1% had completed high school or less. Most participants lived in multi-person households (94.3%), and 5.7% lived alone. Household income comprised 36.5% in the upper quartile, 33.3% in the upper-middle, 22.6% in the lower-middle, and 7.6% in the lowest quartile.

As for lifestyle and behavioural factors, 14.5% reported lifetime smoking experience and 5.7% reported heavy episodic drinking. Only 19.6% reported engaging in muscle-strengthening exercise on at least one day per week. Regarding perceived health, 31.5% rated their health as good, 53.1% as average, and 15.4% as poor. Perceived body size varied; 49.3% perceived themselves as obese, 39.9% as average, and 10.8% as thin.

The mean PHQ-9 score was 3.00 (±3.75), and 24.1% (95% CI 22.8–25.3) scored 5 or above, indicating mild or greater depressive symptoms; 6.4% (95% CI 5.7–7.0) scored 10 or above. A further 31.8% reported high levels of perceived stress. Mean systolic and diastolic blood pressures were 110.1 mmHg and 73.1 mmHg, respectively. Mean HDL cholesterol was 56.6 mg/dL, triglycerides averaged 104.8 mg/dL, mean fasting blood sugar was 94.4 mg/dL, and mean HbA1c was 5.50%.

### 3.2. Differences in Variables Between Women with and Without Elevated Depressive Symptoms

Among the 7595 women in the analytic sample, 1738 were classified as having mild-or-greater depressive symptoms on the basis of a PHQ-9 score of 5 or above, corresponding to a weighted prevalence of 24.1% (Table 2). Significant differences were observed between the groups with and without elevated depressive symptoms across various sociodemographic, behavioral, psychological, and metabolic factors.

The group with depression was significantly younger (mean age 39 ± 11 years) than the group without elevated depressive symptoms (42 ± 11 years) (*p* < 0.001). Educational level had a marginal association; those with a high school education or less had a higher proportion of depressive symptoms (*p* = 0.0502). Income level was significantly associated with depression; 12.0% of the group with depression were in the lowest income quartile, whereas 30.0% were in the highest (*p* < 0.001). Those in single-person households also had higher rates of depression (8.3%) compared to those in multi-person households (3.5%) (*p* < 0.001). No significant differences were observed in depression prevalence by residence area.

In the group with elevated depressive symptoms, 25.9% reported lifetime smoking experience and 10.9% reported heavy episodic drinking, compared with 10.9% and 4.1%, respectively, in the group without elevated symptoms (both *p* < 0.001). Muscle-strengthening exercise did not differ significantly between the two groups.

Approximately 32.4% of those with elevated depressive symptoms rated their health as “bad,” whereas only 10.0% of those without did (*p* < 0.001). Only 15.9% of the group with elevated symptoms rated their health as “good,” compared with 36.5% of the group without. A higher proportion of those with elevated symptoms perceived themselves as obese (53.1%) than of those without (48.1%) (*p* < 0.001).

Among participants with elevated depressive symptoms, 64.7% reported high stress, whereas only 21.4% of those without did (*p* < 0.001).

Women with mild-or-greater depressive symptoms had slightly lower systolic blood pressure (108.9 ± 13.7 mmHg vs. 110.4 ± 14.0 mmHg; *p* < 0.001), slightly lower diastolic blood pressure (72.6 ± 9.7 vs. 73.2 ± 9.3 mmHg; *p* = 0.030), higher triglycerides (109.6 ± 90.4 mg/dL vs. 103.3 ± 79.4 mg/dL; *p* = 0.014), and marginally lower HbA1c (5.46 ± 0.60% vs. 5.51 ± 0.63%; *p* = 0.006) than those without elevated symptoms. No significant differences were observed in HDL cholesterol or fasting blood glucose. Given that the absolute difference in systolic blood pressure is 1 mmHg, this difference is statistically detectable in a sample of this size but is not clinically meaningful.

### 3.3. Factors Associated with Depression Among Korean Women

To examine the factors associated with elevated depressive symptoms among Korean women, logistic regression analyses were conducted by age group (20–39 years and 40–59 years) using 3 sequential models (Table 3).

Model 1 adjusted for demographic characteristics. Lower education and lower household income were associated with higher odds of elevated depressive symptoms, though the strength of each association differed by age group. Compared with those holding a college degree or higher, women with less education had higher odds of elevated depressive symptoms in both age groups (20–39 years: β = 0.17, *p* = 0.002; 40–59 years: β = 0.11, *p* = 0.041). Similarly, compared to women in the upper income quartile, those in lower income quartiles showed significantly higher odds of elevated depressive symptoms, with a stronger association in the older age group (20–39 years: OR 1.11, 95% CI 1.00–1.22; 40–59 years: OR 1.38, 95% CI 1.26–1.52). Notably, living in a single-person household was a significant correlate of elevated depressive symptoms among women aged 40–59 years (β = 0.53, *p* = 0.001), whereas no significant effect was found in the younger age group.

In Model 2, the addition of lifestyle and psychological factors revealed several significant associations. Lifetime smoking experience emerged as a strong correlate of elevated depressive symptoms in both age groups (20–39 years: β = 0.55, *p* < 0.001; 40–59 years: β = 0.59, *p* < 0.001). Heavy episodic drinking was associated with elevated depressive symptoms among younger women (β = 0.59, *p* < 0.001) but not among midlife women (β = 0.37, *p* = 0.235).

Subjective health status was also a critical factor. Women who perceived their health as poor reporting higher levels of depressive symptoms compared to those who rated their health as good (20–39 years: β = 0.75, *p* < 0.001; 40–59 years: β = 0.94, *p* < 0.001). Among all variables, stress perception demonstrated the strongest association with depression. Women who reported high stress were significantly more likely to experience depressive symptoms (20–39 years: β = 1.64, *p* < 0.001; 40–59 years: β = 1.60, *p* < 0.001). Muscle-strengthening exercise showed a modest positive association with elevated depressive symptoms among women aged 20–39 years (OR 1.29, 95% CI 1.01–1.65, *p* = 0.042) but was not associated among women aged 40–59 years (OR 0.91, 95% CI 0.70–1.20, *p* = 0.517). This association was sensitive to the operational threshold: under the national two-days-per-week criterion the estimate was of similar magnitude but no longer significant in the younger group (OR 1.29, 95% CI 0.97–1.70, *p* = 0.076).

Model 3 incorporated perceived body size into the analyses to assess whether it retained an independent association with elevated depressive symptoms after adjustment for all previous variables. Perceived body size was entered as indicator contrasts, with average body size as the reference, so that the thin and obese categories could be examined separately. Neither category was associated with elevated depressive symptoms in either age group (20–39 years, thin: OR 1.25, 95% CI 0.92–1.69, *p* = 0.159; obese: OR 1.05, 95% CI 0.85–1.30, *p* = 0.629; 40–59 years, thin: OR 1.21, 95% CI 0.84–1.73, *p* = 0.311; obese: OR 0.99, 95% CI 0.80–1.22, *p* = 0.887), and the joint two-degree-of-freedom Wald test was likewise non-significant (20–39 years: F(2, 585) = 0.99, *p* = 0.371; 40–59 years: F(2, 633) = 0.69, *p* = 0.500). The near-zero coefficients therefore do not reflect a non-monotonic association obscured by linear parameterisation.

### 3.4. Predictive Performance of Depression Models by Age Group

To assess the predictive validity of logistic regression models for elevated depressive symptoms among Korean women, the 3 models were evaluated across 2 age groups (20–39 and 40–59 years) using classification metrics including accuracy, sensitivity, specificity, and AUC (Table 4).

In the age group 20–39 years, Model 1 demonstrated limited predictive utility, with 54.6% accuracy and an AUC of 0.553, indicating poor discrimination between women with and without elevated depressive symptoms. The inclusion of behavioural and psychological variables in Model 2 substantially improved performance, raising accuracy to 73.7% and the AUC to 0.787. Sensitivity rose from 50.6% in Model 1 to 70.6% in Model 2 and specificity from 56.3% to 75.0%, indicating that lifestyle and mental health variables carried most of the discriminatory information for younger women.

Adding perceived body size in Model 3 did not improve discrimination: the AUC was essentially unchanged (0.786 vs. 0.787 in Model 2), and accuracy (73.7%) and specificity (74.9%) were virtually identical while sensitivity shifted slightly (to 70.8%). These negligible differences are consistent with the non-significant regression coefficients for perceived body size and indicate that it did not contribute meaningfully to model performance in this age group.

The pattern in the 40–59-year-old group was similar. Model 1 again yielded modest performance (accuracy 53.1%, AUC 0.597), while Model 2 achieved substantially better discrimination (accuracy 74.0%, AUC 0.791). Adding perceived body size in Model 3 left performance essentially unchanged (accuracy 73.9%, AUC 0.791).

Overall, the AUC values of both Model 2 and Model 3 in each age group approached 0.79, reflecting acceptable classification performance. Because all three models were estimated on the same analytic sample, the sequential comparison is not confounded by a change in sample composition. The negligible difference between Models 2 and 3—an AUC change of 0.001 or less in both age groups—indicates that perceived body size contributed no discriminative value once behavioural and psychological factors were accounted for. Four sensitivity analyses were conducted. First, redefining the outcome with the more stringent PHQ-9 cut-off of 10 or above, corresponding to the national indicator for depressive disorder, reproduced the pattern reported above: perceived stress, subjective health, lifetime smoking, and heavy episodic drinking remained strongly associated with the outcome, whereas neither perceived body size category was associated in either age group (20–39 years, thin: OR 1.52, 95% CI 0.90–2.55; obese: OR 1.08, 95% CI 0.76–1.53; 40–59 years, thin: OR 0.79, 95% CI 0.41–1.54; obese: OR 1.11, 95% CI 0.74–1.66). Second, modelling the PHQ-9 total score as a continuous outcome, which retains the graded information lost by dichotomisation, showed a small association with perceived thinness among younger women (b = 0.39, *p* = 0.046) but not among midlife women (b = 0.28, *p* = 0.100), and no association with perceived obesity in either group (20–39 years: b = 0.07, *p* = 0.639; 40–59 years: b = 0.05, *p* = 0.622); perceived stress and subjective health remained the dominant correlates throughout. Third, a pooled model incorporating age-group interaction terms showed evidence of effect modification for muscle-strengthening exercise (β = 0.37, *p* = 0.047) and a non-significant trend for household income (β = −0.14, *p* = 0.060); the interactions for single-person households, heavy episodic drinking, and perceived body size were not significant, the last on a joint two-degree-of-freedom test (F = 0.02, *p* = 0.976). Fourth, re-specifying muscle-strengthening exercise using the national two-days-per-week criterion yielded an estimate of similar magnitude that no longer reached significance among younger women (OR 1.29, 95% CI 0.97–1.70, *p* = 0.076).

## 4. Discussion

This study examined the sociodemographic, behavioral, and psychological factors associated with depression among Korean women aged 20–59 years, analyzed separately for those in their 20–30 s and 40–50 s, and evaluated whether subjective body shape perception retained an independent association after adjustment. Approximately 23% of the participants reported depressive symptoms and had a PHQ-9 score ≥ 5; significant differences were found between the women with and without elevated depressive symptoms in key health-related behaviors including smoking, heavy episodic drinking, perceived health status, and perceived stress. Moreover, perceived body size was not associated with depressive symptoms in either age group, although the relative prominence of other correlates differed between them, indicating that the underlying factors associated with depression differed across generations.

Two interpretive constraints frame the discussion that follows. First, our outcome should be understood as elevated depressive symptomatology at or above the mild threshold (PHQ-9 ≥ 5), not as diagnosed or probable major depressive disorder; the 24.1% figure is accordingly not comparable with prevalence estimates derived from the ≥ 10 screening threshold, which yielded 6.4% in the same sample, or from diagnostic interview. Second, because the complex survey design was incorporated, the estimates refer to the national population of Korean women aged 20–59 years, although they describe an average over the four pooled cycles rather than any single year.

Depression was more prevalent among the participants in their 20 s and 30 s, whereas the rate was relatively lower among those in their 40 s and 50 s. This age gradient is consistent with global reports of a rising burden of depressive symptoms among adolescent females and young women [16]. We note, however, that our pooled cross-sectional design observes a single aggregated time point and therefore cannot itself establish change over time; the secular trend is offered here as background context rather than as a finding of the present study. Demonstrating such a change requires a repeated cross-sectional design holding the depressive-symptom measure comparable across two or more time points, as applied to young women elsewhere [17]. Young women are not only more emotionally vulnerable but also situated in a transitional phase marked by academic demands, employment challenges, and expanding social roles [18]. They are particularly susceptible to sociocultural pressures centered on appearance, peer comparison, and social media exposure [16,19]. Recent studies have shown that Instagram use among adolescent and young women often leads to repeated comparisons with idealized body images of others, which can elevate levels of depression through the mediating effects of body dissatisfaction and low self-esteem [20]. Consistent with this context, in unadjusted comparisons a higher proportion of women in their 20 s and 30 s with depressive symptoms perceived themselves as overweight, even when this perception did not correspond to their actual BMI. However, this bivariate association did not persist after adjustment: subjective body perception was not independently associated with depression in either age group. The interpretation of this attenuation, however, is not straightforward, and we consider two readings that the present data cannot adjudicate between. Under the first, perceived stress and subjective health are prior characteristics that generate both a more negative appraisal of body size and a greater burden of depressive symptoms, so that the crude association is confounded and adjustment removes a spurious signal. Under the second, appraising one’s body unfavorably itself elevates perceived stress and depresses self-rated health, which in turn relate to depressive symptoms; on this reading the covariates lie on the causal path, conditioning on them constitutes overadjustment, and the attenuation reflects mediation rather than confounding. The second reading has substantial theoretical support, since body dissatisfaction is widely held to elevate distress and to worsen self-rated health. We therefore do not claim that perceived body size has no role in depressive symptoms. What the data establish is narrower: perceived body size carries no information about depressive symptoms beyond what perceived stress and subjective health already convey. Whether it operates through them, or is merely correlated with them, requires a longitudinal design with measured temporal ordering, or a formal mediation analysis with explicit identifying assumptions, neither of which is available here.

Kuehner [19] identified several factors contributing to the higher prevalence of depression in women, including body image dissatisfaction, body shame, rumination, and a tendency toward social comparison. Kuehner [19] further explained that these affective and cognitive tendencies tend to intensify after adolescence, increasing women’s sensitivity to stress and emotional reactivity [19]. These theoretical accounts position body image as relevant to women’s emotional health, and are in fact more consistent with the mediation reading outlined above than with the confounding reading, since they describe body dissatisfaction as generating distress rather than as arising from it. Our adjusted results are therefore best read not as evidence against the clinical relevance of body image, but as evidence that its statistical association with depressive symptoms is not separable from perceived stress and self-rated health in cross-sectional data of this kind. Our participants with depression also exhibited distinct differences in health-related characteristics. Compared to those without elevated depressive symptoms, they reported more negative perceptions of their overall health, higher levels of perceived stress, and significantly greater engagement in smoking and heavy episodic drinking. These findings suggest that emotional dysregulation may influence maladaptive health behaviors, and the interplay of psychological and behavioral factors may serve as a pathway that elevates risk of depressive symptoms [21]. In a large-scale analysis of premenopausal and postmenopausal women in the Republic of Korea, Kim et al. [22] reported that smoking and excessive alcohol consumption significantly increased depression risk, with this pattern being more pronounced among premenopausal women. Notably, women who consumed more than 14 drinks per day or smoked more than 20 cigarettes daily had the highest depression risk [22]. In this context, smoking and alcohol consumption under stress may not merely reflect habitual behaviors, but rather serve as coping strategies for emotional regulation.

After adjustment, subjective body shape perception was not independently associated with depression in either age group. Instead, in midlife women, lifestyle-related factors such as income level, household composition, perceived health status, and perceived stress are more influential [23,24,25,26], suggesting that depression in this population may be more closely related to health concerns, role transitions, and social isolation than to perceived body size or self-perception. Similarly, Luo et al. [27] found that among middle-aged women, obesity status and body shape perception were not significantly associated with depression, whereas perceived health status and socioeconomic conditions demonstrated greater explanatory power.

These midlife findings warrant a more explicit reading. Lower household income is not an appraisal or a behaviour; it is a material condition, and its prominence in midlife points toward economic insecurity as a circumstance under which depressive symptoms become more probable. Living alone showed the same pattern in the demographic model (OR 1.70, 95% CI 1.24–2.33) but was attenuated to non-significance once behavioural and psychological factors were added, which suggests that its association with depressive symptoms operates largely through the stress and perceived-health pathways rather than independently of them. Framing the response to such findings primarily in terms of stress reduction, health management, and screening anchored in perceived health would place the explanatory and interventional burden on individual conduct while the structural correlates our own analysis surfaces recede into the background. We note this tension rather than resolve it. Income adequacy and household composition are not modifiable by clinical encounter, and screening that identifies a midlife woman living alone on a low income as at elevated risk of depressive symptoms does not thereby supply what that risk arises from. Individual-level intervention and structural policy address different points in the same causal structure, and the findings reported here bear on both.

In both age groups, model discrimination was driven by behavioral and psychological factors, and the addition of subjective body shape perception did not materially change predictive performance. Nonetheless, the relative contribution of individual determinants differed by age: heavy episodic drinking was associated with depression mainly among younger women, whereas lower income and single-person households were more salient in midlife. These differences support age-specific screening approaches that prioritize psychological distress and perceived health rather than perceived body size. A further behavioural finding warrants comment. Among women aged 20–39 years, those reporting muscle-strengthening exercise had somewhat higher odds of elevated depressive symptoms. Two considerations temper this observation. First, the association was sensitive to how the exposure was operationalised, weakening to non-significance under the national two-days-per-week criterion, so it should be regarded as provisional. Second, in a cross-sectional design the direction of the relationship cannot be established. Resistance training among young Korean women is frequently undertaken for weight and body-shape management rather than for general fitness, so appearance-related concerns may plausibly drive both exercise participation and depressive symptoms, and reverse causation cannot be excluded. Prospective designs that distinguish the motivation for exercise from its frequency would be needed to clarify this pattern.

These findings bear on the tailoring of mental health provision by age group. For younger women, attention to perceived stress and psychological distress, alongside appearance-related pressures where clinically indicated, appears most relevant. For midlife women, individual-level provision addressing stress and health management is likely to be necessary but not sufficient, since the correlates most prominent in this group concern income adequacy and household composition. Policies addressing economic security and social connectedness in midlife therefore sit alongside, rather than downstream of, clinical provision.

This study has several limitations. First, due to its cross-sectional design, causal relationships between variables could not be established. The association between depression and health behaviors may involve reciprocal influences; thus, longitudinal studies are needed to better understand the directionality of these relationships. Second, perceived body size was assessed with a single categorical item that captures appraisal of body size only. It does not measure body dissatisfaction, appearance-related distress, or the affective and evaluative dimensions of body image that the broader literature implicates in depression, and our null finding therefore speaks to this narrow appraisal measure and not to body image as a construct. A study employing a validated multidimensional instrument might well reach a different conclusion. Third, although this study controlled for sociodemographic and health-related factors, it did not include other potentially influential psychosocial variables such as social support, life events, and mental health history. Future studies should incorporate a more comprehensive range of covariates to enhance explanatory power. Fourth, the KNHANES employs a stratified, multistage clustered probability sampling design with post-stratification weights, and these design elements were incorporated into the present analyses. Integrated sampling weights, variance-estimation strata, and primary sampling units were specified, and variances were estimated by Taylor-series linearisation, so that the estimates reported here refer to the population of Korean women aged 20–59 years rather than to the analytic sample alone. Two qualifications nonetheless remain. Design-based estimation adjusts for unequal selection probabilities and for unit non-response through the weighting scheme, but it cannot correct for residual differences between respondents and non-respondents on characteristics unrelated to the weighting variables. Furthermore, because four survey cycles were pooled into a single cross-section, the estimates describe an average over the pooled period rather than any individual year, and secular change across cycles is absorbed rather than modelled. Fifth, elevated depressive symptomatology was defined using a PHQ-9 cut-off of ≥ 5, which captures mild or greater depressive symptoms and is more inclusive than the ≥ 10 threshold used to screen for probable major depression; the 23% figure therefore reflects the prevalence of at least mild depressive symptoms rather than probable major depressive disorder. Sixth, the heavy episodic drinking item is administered only to respondents who report consuming alcohol, so records for lifetime and past-year abstainers are structurally skipped rather than missing. These respondents were classified as not engaging in heavy episodic drinking, which allowed all three models to be estimated on a single analytic sample and removed the differential attrition that listwise deletion on this item would otherwise have introduced. The measure nonetheless rests on self-report and on a threshold of ten or more drinks on a single occasion, which is more stringent than the conventional criterion applied to women; the prevalence of heavy episodic drinking reported here is therefore conservative, and the corresponding coefficients may be attenuated relative to estimates based on the standard threshold. Seventh, a difference in statistical significance between separately fitted models does not by itself establish that an association differs across subgroups. We therefore fitted a pooled model incorporating age-group interaction terms for the demographic, behavioural, and body size variables. Household income showed only a non-significant trend towards effect modification (β = −0.14, *p* = 0.060), with the association stronger among midlife women, and muscle-strengthening exercise showed a marginal interaction (β = 0.37, *p* = 0.047); the interaction terms for single-person households, heavy episodic drinking, and perceived body size were all far from significance, the last on a joint two-degree-of-freedom test (F = 0.02, *p* = 0.976). The age-stratified results are accordingly presented as descriptive life-stage patterns rather than as demonstrated differences. Interaction tests of this kind have limited statistical power, so genuine but smaller differences may have gone undetected. Eighth, muscle-strengthening exercise was measured by a single item on weekly frequency, with no information on intensity, duration, or the motivation for participation, and was dichotomised at one day per week. The association observed among younger women did not persist under the alternative two-days-per-week threshold and should therefore be interpreted with caution.

## 5. Conclusions

This study provides several academic and practical implications. First, using pooled data from a large national health survey, age-group differences in factors related to depressive symptoms among women were empirically examined, integrating health behaviors and psychological perceptions. Second, the prediction models provided a foundation for age-specific screening strategies. Third, by examining perceived body size alongside behavioral and psychological factors, this study shows that its crude association with depressive symptoms does not persist after adjustment, and that perceived stress and subjective health are the characteristics most strongly associated with depressive symptoms in this sample. Whether perceived body size operates through these characteristics or is merely correlated with them cannot be determined from cross-sectional data, and this question is a priority for longitudinal work. Fourth, the prominence of household income and household composition among midlife women indicates that the conditions associated with depressive symptoms in this group are partly structural, and that symptom-level screening, while useful for identification, does not by itself address them.

## Figures and Tables

**Table 1:** General Characteristics of the Analytic Sample (N = 7595), Weighted for the Complex Survey Design.

Variables	Women (N = 7595) Weighted % (Unweighted n)
Demographic Factors	
Age in years	
20–29	22.3 (1359)
30–39	24.5 (1935)
40–49	27.9 (2206)
50–59	25.3 (2095)
Residence area	
City	88.1 (6534)
Rural	11.9 (1061)
Education level	
High school or lower	40.1 (3110)
Associate degree	20.3 (1534)
College or higher	39.6 (2951)
Income quartile (household)	
Lower	7.6 (536)
Lower middle	22.6 (1726)
Upper middle	33.3 (2522)
Upper	36.5 (2811)
Household generation	
One person household	5.7 (437)
Non-single-person household	94.3 (7158)
Lifestyle and Behavioral Factors	
Lifetime smoking experience	
Ever smoked	14.5 (1052)
Never smoked	85.5 (6543)
Heavy drinking	
No	94.3 (7212)
Yes	5.7 (383)
Muscle-strengthening exercise	
Yes	19.6 (1476)
No	80.4 (6118)
Subjective health status	
Bad	15.4 (1151)
Average	53.1 (4058)
Good	31.5 (2386)
Subjective body perception	
Thin	10.8 (822)
Average	39.9 (3044)
Obese	49.3 (3729)
Body mass index	
Underweight	7.0 (515)
Normal	51.3 (3867)
Obese	41.7 (3213)
Mental and Psychological Health Status	
Patient Health Questionnaire-9	
	3.00 ± 3.75
0–4	75.9 (5857)
≥5	24.1 (1738)
Stress perception	
Feel less	68.2 (5202)
Feel a lot	31.8 (2393)
Biochemical Markers	
Systolic blood pressure (mmHg)	
	110.1 ± 14.0
Diastolic blood pressure (mmHg)	
	73.1 ± 9.4
High-density lipoprotein cholesterol (mg/dL)	
	56.6 ± 12.6
Triglycerides (mg/dL)	
	104.8 ± 82.2
Fasting blood sugar (mg/dL)	
	94.4 ± 17.9
Glycated hemoglobin (%)	
	5.50 ± 0.62

**Table 2:** Group Differences in Health-Related Characteristics by PHQ-9 Score Among Women (N = 7595), Weighted for the Complex Survey Design.

Variables	PHQ-9: 0–4 (*n* = 5857) Weighted % (n) or M ± SD	PHQ-9: ≥5 (*n* = 1738) Weighted % (n) or M ± SD	t/F (df)	*p*-Value
Demographic Factors				
Age in years	41.0 ± 11.1	37.6 ± 11.4	1641 (91.01)	<0.001 ***
20–29	19.5 (917)	30.9 (442)		
30–39	23.7 (1438)	27.3 (497)		
40–49	30.0 (1800)	21.6 (406)		
50–59	26.9 (1702)	20.2 (393)		
Residence area			1641 (0.97)	0.325
City	88.3 (5049)	87.2 (1485)		
Rural	11.7 (808)	12.8 (253)		
Education level			2640 (2.32)	0.099
High school or lower	40.1 (2385)	40.1 (725)		
Associate degree	19.7 (1157)	22.1 (377)		
College or higher	40.2 (2315)	37.8 (636)		
Income quartile (household)			3639 (19.51)	<0.001 ***
Lower	6.3 (333)	12.0 (203)		
Lower middle	21.7 (1282)	25.3 (444)		
Upper middle	33.7 (1956)	32.3 (566)		
Upper	38.4 (2286)	30.5 (525)		
Household generation			1641 (22.65)	<0.001 ***
One person household	5.0 (292)	7.9 (145)		
Non-single-person Household	95.0 (5565)	92.1 (1593)		
Lifestyle and Behavioral Factors				
Lifetime smoking experience			1641 (168.45)	<0.001 ***
Ever smoked	10.9 (622)	25.9 (430)		
Never smoked	89.1 (5235)	74.1 (1308)		
Heavy episodic drinking			1633 (68.57)	<0.001 ***
No	95.9 (4163)	89.1 (1179)		
Yes	4.1 (222)	10.9 (161)		
Muscle-strengthening exercise			1641 (0.00)	0.964
Yes	19.6 (1161)	19.5 (315)		
No	80.4 (4695)	80.5 (1423)		
Subjective health status			2640 (191.85)	<0.001 ***
Bad	10.0 (587)	32.4 (564)		
Average	53.5 (3134)	51.6 (924)		
Good	36.5 (2136)	15.9 (250)		
Subjective body perception			2640 (9.63)	<0.001 ***
Thin	10.4 (606)	12.1 (216)		
Average	41.5 (2444)	34.8 (600)		
Obese	48.1 (2807)	53.1 (922)		
Mental and Psychological Health Status				
Perceived stress			1641 (876.04)	<0.001 ***
Feel less	78.6 (4607)	35.3 (595)		
Feel a lot	21.4 (1250)	64.7 (1143)		
Biochemical Markers				
Systolic blood pressure (mmHg)			−3.79	<0.001 ***
	110.4 ± 14.0	108.9 ± 13.7		
Diastolic blood pressure (mmHg)			−2.18	0.030 *
	73.2 ± 9.3	72.6 ± 9.7		
High-density lipoprotein cholesterol (mg/dL)			0.90	0.366
	56.6 ± 12.6	56.9 ± 12.6		
Triglycerides (mg/dL)			2.46	0.014 *
	103.3 ± 79.4	109.6 ± 90.4		
Fasting blood sugar (mg/dL)			−1.72	0.087
	94.6 ± 18.0	93.7 ± 17.4		
Glycated hemoglobin (%)			−2.74	0.006 **
	5.51 ± 0.63	5.46 ± 0.60		

* *p* < 0.05, ** *p* < 0.01, *** *p* < 0.001. M, mean; PHQ-9, Patient Health Questionnaire; SD, standard deviation.

**Table 3:** Logistic Regression Analysis of Factors Associated with Mild-or-Greater Depressive Symptoms (PHQ-9 ≥ 5).

Variables	Age 20–39 Years					Age 40–59 Years				
	Coefficient	Std. Error	z Value	*p*-Value	OR (95% CI)	Coefficient	Std. Error	z Value	*p*-Value	OR (95% CI)
MODEL 1 (N = 7595)										
Area of residence	0.06	0.15	0.38	0.7020	1.06 (0.79–1.41)	0.05	0.12	0.38	0.7080	1.05 (0.82–1.34)
Education level	0.17	0.06	3.06	0.0023 **	1.19 (1.06–1.33)	0.11	0.05	2.05	0.0405 *	1.11 (1.01–1.23)
Income quartile (household)	0.10	0.05	2.02	0.0442 *	1.11 (1.00–1.22)	0.32	0.05	6.70	<0.001 ***	1.38 (1.26–1.52)
Household generation	0.26	0.16	1.64	0.1019	1.29 (0.95–1.75)	0.53	0.16	3.31	0.0010 **	1.70 (1.24–2.33)
MODEL 2 (N = 7594)										
Area of residence	0.07	0.15	0.49	0.6220	1.08 (0.80–1.45)	0.02	0.14	0.16	0.8710	1.02 (0.78–1.35)
Education level	−0.01	0.06	−0.21	0.8320	0.99 (0.87–1.12)	0.07	0.06	1.04	0.2970	1.07 (0.94–1.21)
Income quartile (household)	0.08	0.06	1.33	0.1850	1.08 (0.96–1.21)	0.20	0.05	3.66	<0.001 ***	1.22 (1.10–1.36)
Household generation	0.13	0.20	0.62	0.5370	1.13 (0.76–1.69)	0.31	0.19	1.63	0.1030	1.36 (0.94–1.97)
Lifetime smoking experience	0.55	0.13	4.34	<0.001 ***	1.74 (1.35–2.23)	0.59	0.15	3.92	<0.001 ***	1.80 (1.34–2.41)
Heavy episodic drinking	0.59	0.17	3.50	<0.001 ***	1.79 (1.29–2.49)	0.37	0.31	1.19	0.2350	1.45 (0.79–2.66)
Muscle-strengthening exercise	0.25	0.13	2.01	0.0447 *	1.29 (1.01–1.64)	−0.09	0.14	−0.62	0.5360	0.92 (0.70–1.20)
Subjective health status	0.75	0.09	8.59	<0.001 ***	2.11 (1.78–2.50)	0.94	0.08	11.73	<0.001 ***	2.56 (2.19–2.99)
Perceived stress	1.64	0.10	16.54	<0.001 ***	5.16 (4.25–6.27)	1.60	0.10	16.08	<0.001 ***	4.94 (4.06–6.00)
MODEL 3 (N = 7594)										
Area of residence	0.08	0.15	0.50	0.6160	1.08 (0.80–1.45)	0.02	0.14	0.15	0.8800	1.02 (0.77–1.35)
Education level	−0.01	0.06	−0.17	0.8680	0.99 (0.87–1.12)	0.07	0.06	1.10	0.2730	1.07 (0.95–1.22)
Income quartile (household)	0.08	0.06	1.36	0.1740	1.08 (0.97–1.21)	0.20	0.05	3.62	<0.001 ***	1.22 (1.09–1.35)
Household generation	0.13	0.21	0.63	0.5280	1.14 (0.76–1.70)	0.31	0.19	1.65	0.0990	1.37 (0.94–1.99)
Lifetime smoking experience	0.56	0.13	4.37	<0.001 ***	1.75 (1.36–2.24)	0.58	0.15	3.88	<0.001 ***	1.79 (1.33–2.41)
Heavy episodic drinking	0.59	0.17	3.46	<0.001 ***	1.80 (1.29–2.51)	0.38	0.31	1.22	0.2230	1.46 (0.79–2.69)
Muscle-strengthening exercise	0.26	0.13	2.04	0.0421 *	1.29 (1.01–1.65)	−0.09	0.14	−0.65	0.5170	0.91 (0.70–1.20)
Subjective health status	0.75	0.09	8.65	<0.001 ***	2.11 (1.78–2.50)	0.94	0.08	11.56	<0.001 ***	2.55 (2.18–3.00)
Perceived stress	1.64	0.10	16.56	<0.001 ***	5.15 (4.24–6.25)	1.60	0.10	16.03	<0.001 ***	4.93 (4.06–6.00)
Perceived body size: thin vs. average	0.22	0.16	1.41	0.1590	1.25 (0.92–1.69)	0.19	0.18	1.02	0.3110	1.21 (0.84–1.73)
Perceived body size: obese vs. average	0.05	0.11	0.48	0.6290	1.05 (0.85–1.30)	−0.02	0.11	−0.14	0.8870	0.99 (0.80–1.22)

* *p* < 0.05, ** *p* < 0.01, *** *p* < 0.001. Std., standard. Model 1: adjusted for demographic factors (area of residence + income quartile [household] + household generation); Model 2: Model 1 + behavioral and psychological factors (current smoking status + heavy episodic drinking + muscle-strengthening exercise + subjective health status + perceived stress); Model 3: Model 2 + perceived body size. Reference categories were city for residence area, college or higher for education, highest quartile for household income, multi-person household for household generation, no lifetime smoking for smoking, no for heavy episodic drinking, no for muscle-strengthening exercise, good for subjective health status, low or moderate for perceived stress, and average for perceived body size. Perceived body size was entered as indicator contrasts (thin vs. average and obese vs. average); the joint two-degree-of-freedom Wald test was non-significant in both age groups (20–39 years: F(2, 578) = 0.76, *p* = 0.467; 40–59 years: F(2, 633) = 0.41, *p* = 0.664). All estimates incorporate the KNHANES complex sampling design (integrated weights, variance-estimation strata, and primary sampling units) with Taylor-series linearisation.

**Table 4:** Logistic Regression Models for Predicting Mild-or-Greater Depressive Symptoms (PHQ-9 ≥ 5) in Women: Performance Evaluation.

Model	Age 20–39 Years				Age 40–59 Years			
	Accuracy (%)	Sensitivity (%)	Specificity (%)	AUC	Accuracy (%)	Sensitivity (%)	Specificity (%)	AUC
Model 1	0.5456	0.5059	0.5626	0.5530	0.5312	0.6008	0.5150	0.5974
Model 2	0.7366	0.7060	0.7497	0.7867	0.7398	0.6771	0.7544	0.7911
Model 3	0.7367	0.7083	0.7488	0.7862	0.7392	0.6831	0.7523	0.7913

Model 1: demographic factor adjusted (area of residence + income quartile (household) + household generation); Model 2: Model 1 + life and behavior factor adjusted (current smoking status + alcohol consumption + muscle-strengthening exercise + subjective health status); and Model 3: Model 2 + subjective body perception. AUC, area under the receiver operating characteristic curve.

## Data Availability

Publicly available datasets were analyzed in this study. The Korea National Health and Nutrition Examination Survey raw data can be downloaded from the Korea Disease Control and Prevention Agency at https://knhanes.kdca.go.kr, subject to registration and agreement to the data use terms. No restrictions apply beyond those imposed by the data provider.

## Abbreviations

PHQ-9: Patient Health Questionnaire-9; BMI: Body Mass Index; HDL: High-Density Lipoprotein; HbA1c: Glycated Hemoglobin; KNHANES: Korea National Health and Nutrition Examination Survey; AUC: Area Under the Receiver Operating Characteristic Curve; IRB: Institutional Review Board.
